# MRM-based LC-MS method for accurate C-peptide quantitation

**DOI:** 10.1016/j.jmsacl.2025.02.001

**Published:** 2025-02-12

**Authors:** Will Grothoff, Ivan Khodakivskyi, Aleks Shin, Randie Little, Shawn Connolly, Kuanysh Kabytaev

**Affiliations:** Pathology & Anatomical Sciences, University of Missouri, 1 Hospital Drive, Columbia, MO 65211, USA

**Keywords:** C-peptide, Standardization, LC-MS, Diabetes mellites

## Abstract

**Introduction:**

C-peptide secretion mirrors beta-cell function and has emerged as a valuable clinical biomarker for diabetes mellitus. C-peptide measurements can provide estimates of insulin secretory capacity, aiding in clinical decision-making and differentiation between diabetes types. Unfortunately, C-peptide assays are still not standardized, which may limit their practical clinical use. We have developed an MRM-based LC-MS method that demonstrated accuracy close to our reference method.

**Objective:**

To develop and validate a mass spectrometry method for accurate quantitation of C-peptide.

**Method:**

A serum sample was spiked with isotope-labeled C-peptide as a standard. The enrichment process involved protein precipitation with methanol, solid-phase extraction, and anion exchange for C-peptide enrichment followed by Glu-C digestion. The peptide LGGGPGAGSLQPLALE was quantitated using MRM in positive ion mode. The calibration process includes C-peptide CRM material to ensure a complete traceability chain for the measurement.

**Results:**

The assay exhibited linearity across a wide range of C-peptide concentrations and a limit of quantitation of 0.058 nmol/L. The inter-day imprecision was less than 9.6 % CV, and the intra-day imprecision was less than 8.9 % CV. Spiking with bilirubin, triglycerides, and hemoglobin demonstrated no interference, except for triglycerides at very high levels. The method exhibited a strong correlation to the C-peptide reference method (r2 = 0.95).

**Conclusion:**

The developed mass spectrometry method has demonstrated accurate results in C-peptide quantitation and can serve as a supplemental method to the existing C-peptide reference method. This ensures sustained stability over time and ultimately refines the existing reference system.

## Highlights

• C-peptide is a proxy for insulin secretion, attracting growing clinical interest.
• C-peptide assay variability poses a challenge for diabetes management.
• We developed an LC-MS/MS method that can complement the C-peptide reference method.

## Introduction

C-peptide and insulin are both secreted in equimolar concentrations by the pancreas. Subsequently, insulin undergoes hepatic clearance before being extracted by insulin-sensitive tissues, such as skeletal muscle, renal tissue, and adipose tissue [1]. In contrast, C-peptide is minimally affected by these processes. As a result, C-peptide has a higher concentration and a longer half-life in the blood compared to insulin, making it a convenient proxy for assessing insulin production. The test holds promise for diabetes management, as evidence has demonstrated that even residual C-peptide secretion has clinical benefits for individuals with type 1 diabetes [2], [3], [4]. Additionally, the measurement of C-peptide may provide clinical advantages for individuals with type 2 diabetes [5]. As type 1 and type 2 diabetes manifest differences in insulin secretion, C-peptide can aid in distinguishing between diabetes types, helping to avoid misclassification [6], [7].

The variability in commercial assays poses a hurdle to the expanded usefulness of C-peptide in clinical applications [8]. This lack of standardization or harmonization presents a challenge to the efficient management of patients [9]. For medical laboratory tests, it is crucial to ensure that C-peptide results are consistent and comparable to avoid diagnostic inaccuracies, unsuitable treatments, and unreliable patient monitoring. This lack of comparability can also undermine clinical research. For example, in *meta*-analyses or systematic reviews, disparate results make drawing conclusions challenging. Such issues impede the generation of clear evidence, the drafting of recommendations, and the establishment of consensus on definitions and diagnostic cut points.

To address this issue, we previously developed a reference method for the quantitation of intact C-peptide using liquid chromatography–mass spectrometry (LC-MS), calibrated by a Certified Reference Material (CRM) from the National Metrology Institute of Japan (NMIJ CRM 6901-b), which is currently listed in the Joint Committee for Traceability in Laboratory Medicine (JCTLM) [10], [11], [12], [13], [14]. We regularly create serum-based secondary reference materials with C-peptide values assigned by our reference method. This includes a set of 40 samples from both healthy individuals and patients with diabetes, spanning a wide C-peptide range, which we provide to assay vendors for recalibration purposes [15]. Our scheme ensures full traceability and commutability for our reference system. Having a reference method implies regular comparative studies with other research groups and methods to examine potential method instability. Additionally, sharing our secondary reference materials to calibrate laboratory-developed LC-MS C-peptide assays is important for the dissemination of standardized C-peptide testing [12], [14], [16].

While our reference method is specific, employing it with individual specimens in matrix poses a potential risk since the method is based on selected ion monitoring (SIM) quantitation. The risk stems from the possibility that some patient samples within the secondary reference material set may exhibit interference, potentially influencing the overall recalibration curve of the assays. It is crucial to acknowledge that serum is a complex matrix and can vary from one individual to another on the nanomolar scale where C-peptide is present. This means that SIM, which controls only the *m*/*z* of a peptide at Q1, is prone to potential interference. In contrast, Multiple Reaction Monitoring (MRM), which simultaneously controls the *m*/*z* of the peptide at Q1 and its daughter ions at Q3, reduces the likelihood of interference and ensures more accurate quantitation. The current study is dedicated to the development of C-peptide quantitation based on enzymatic digestion followed by MRM quantitation. In our laboratory, we plan to use the developed method as a supplement to the reference method to ensure specificity and long-term stability of the C-peptide reference system. Ultimately, this method could be registered with JCTLM to refine the reference C-peptide system, where MRM completely replaces the somewhat dated SIM methodology.

## Materials and methods

### Reagents and materials

DL-Dithiothreitol, isotopically labeled C-peptide (EAEDLQVGQVELGGGPGAGSLQPLAL(^13^C_6_)EGSL(^13^C_6_)Q), DL-dithiothreitol (DTT), human serum albumin (HSA) and formic acid were obtained from Sigma-Aldrich (St. Louis, MO). Iodoacetamide (IAA), sequence grade Glu-C, horse serum, and acetonitrile were obtained from Fisher Scientific (Pittsburgh, PA). C-peptide (certified reference material, CRM, 6901-b) was obtained from the National Metrology Institute of Japan. EAEDLQVGQVELGGGPGAGSLQPLALEGSLQ was synthesized using solid-phase FMOC chemistry methodology in a core facility (University of Missouri, Columbia, MO). Sep-Pak C18 cartridges (3 cc, 200 mg) were obtained from Waters (Milford, MA). HiTrap Q HP anion exchange columns (1 ml) were obtained from Cytiva (Marlborough, MA). Microcentrifuge tubes (1.5 ml) were obtained from Fisher Scientific (Pittsburgh, PA). Glass tubes (10x75 mm, borosilicate glass) were obtained from Globe Scientific (Mahwah, NJ). The DTT and IAA solutions were freshly prepared. Glu-C was dissolved in water, aliquoted into minimal volumes, and stored at −70 °C. The C-peptide CRM solution and the C-peptide isotope-labeled standard solution were also stored at −70 °C and used only once to avoid freeze–thaw cycles.

### Description of samples

Venous blood was collected from both healthy individuals and diabetic patients at several time points—fasting, and 30 and/or 60 min after consuming a single serving of mixed liquid meal (Boost, Nestle Health Sciences, Vevey, Switzerland)—to establish a broad range of C-peptide levels. The blood was drawn into red top serum tubes (BD Vacutainer®), and following clot formation, it was centrifuged to extract the serum. The serum samples were then frozen and stored at −70 °C. Blood samples were collected (2015–2016) in accordance with IRB approval (University of Missouri, # 2003930) to serve as secondary calibrators for the C-peptide LC-MS reference method. The samples were de-identified, and no identifying information was used in the study. Informed consent was obtained.

### Sample preparation

A 200 μl serum sample was spiked with 20 μl of isotope-labeled standard (concentration: 10^-7^ g/ml in 0.2 % human serum albumin), vortexed, and allowed to equilibrate for 5 min. Subsequently, 1 ml of methanol was added, and the sample was vortexed again. Serum proteins were precipitated by centrifugation at 4000 rpm for 7 min in a microcentrifuge tube. The supernatant was then passed through a Sep-Pak C18 column, which had been pre-equilibrated with 3 ml of aqueous methanol (MeOH:H_2_O = 5:1). To the filtrate, 4 ml of ammonium formate buffer (72 mM, pH 8.33) were added. This solution was then loaded onto an ion-exchange Q column (rate 1 ml/min), which had been pre-equilibrated with 10 ml of working buffer. The column was subsequently eluted with 1 ml of 0.4 % formic acid in water (rate 1 ml/min). The eluted fraction was evaporated to dryness in a glass tube using a centrifugal evaporator at room temperature overnight. The residue was reconstituted with 100 μl of ammonium bicarbonate buffer (0.5 M, pH 8), and 2 μl of Glu-C (0.25 g/ml) was added. The mixture was then incubated at 37 °C for 24 h. Finally, 45 μl of 0.4 % formic acid in water was added before submitting the sample for LC-MS analysis. The Q column was regenerated by washing with 10 ml of 1 M NaCl.

### LC-MS/MS

Mass spectrometry analysis was performed using a QTRAP 6500+ (AB Sciex, Foster City, CA) coupled to a Prominence LC system (Shimadzu, Kyoto, Japan). Chromatographic separation was conducted on a Jupiter C18 column (30 x 4.6 mm, 5 μm, 300 Å, Phenomenex, Torrance, CA). Two mobile phases were used for gradient elution: (A) 0.4 % formic acid in water and (B) 0.4 % formic acid in acetonitrile. The linear gradient was optimized to minimize analysis time while ensuring efficient separation. To prevent carryover, a gradient featuring a double ramp to a pure organic mobile phase was developed and validated by analyzing a sample with a high concentration of C-peptide. For complete assurance in the analysis of very low C-peptide samples, blanks were run beforehand. The gradient profile was as follows (time, %B): 0.1 min, 5 %; 3 min, 5 %; 15 min, 50 %; 20 min, 100 %; 22 min, 100 %; 25 min, 5 %; 30 min, 100 %; 32 min, 5 % with a flow rate of 0.8 ml/min. A switching valve was adjusted to the open position around the targeted retention time to reduce contamination in the mass spectrometer. The column oven temperature was set to 40 °C, and the autosampler temperature was set to 4 °C. Instrument control and data acquisition were performed using Analyst 1.6.3 software (AB Sciex, Foster City, CA), while peak integration was done in MultiQuant (AB Sciex, Foster City, CA). The optimized ESI source parameters were as follows: ion spray voltage 5500 V, temperature (TEM) 250 °C,CUR = GS1 = GS2 = 30 psi, CAD high.

### Assay validation

#### Calibration

A two-point calibration curve was constructed using isotope-labeled C-peptide and certified reference material (from the National Metrology Institute of Japan) as the native C-peptide. The two chosen points were C-peptide solutions in 0.2 % HSA with different molar ratios (1:1 and 1:6) of NMIJ CRM to isotope-labeled C-peptide.

#### Imprecision

To measure intra-day variation, two patient serum samples, with a low concentration of C-peptide (0.53 nmol/L) and a high concentration of C-peptide (2.88 nmol/L), were prepared and analyzed in five replicates. To assess inter-day variation, we prepared and analyzed five replicate aliquots of the two serum samples (low and high) over five consecutive days. One-way ANOVA (Analysis of Variance) was used to calculate intra-day, inter-day, and total CV according to EP15-A3 CLSI.

#### Stability

Two patient serum samples, with a low concentration of C-peptide (0.53 nmol/L) and a high concentration of C-peptide (2.88 nmol/L), were selected for stability testing. Short-term stability of digested samples was examined, considering the length of the LC-MS acquisition queue and the tendency for peptides to degrade in an acidic mobile phase. The serum samples were fractionated and digested following the general protocol (see 2.3), and acidic solutions were stored in the autosampler at 4 °C for 24 h. Serum storage stability was examined by storing the serum samples at room temperature for 24 h and at 4 °C for two days. The percentage of degradation was calculated by comparing the C-peptide concentration of each sample before and after storage.

#### Limit of quantitation

Samples were created by adding C-peptide CRM into horse serum. The serum was initially tested for potential interference and showed no evidence of it. The C-peptide concentrations used were as follows: 0.05 nmol/L, 0.08 nmol/L, 0.10 nmol/L, 0.15 nmol/L, 0.20 nmol/L, 0.25 nmol/L, 0.30 nmol/L, 0.40 nmol/L, and 0.75 nmol/L. The samples were analyzed in triplicate. The lower limit of quantitation was defined as the lowest concentration at which the CV% between replicates is less than 20 %. We found that the signal-to-noise (S/N) criteria are not appropriate for our method, as the S/N ratio decreases too quickly, despite the measured values being accurate based on the spiked amount. This is likely due to the internal algorithms in MultiQuant, where peaks are approximated to an ideal shape.

#### Linearity

To assess the linearity of the method, ten levels of C-peptide spanning a range from 0.4 to 9.5 nmol/L were analyzed. The dilutions were prepared by spiking NMIJ C-peptide CRM into C-peptide deficient serum from a type 1 diabetic individual.

#### Interference

An interference study was conducted by spiking bilirubin, triglycerides, or hemoglobin into pooled patient serum samples with a C-peptide concentration of 1.58 nmol/L. Final concentrations in serum were as follows: bilirubin concentrations up to 43 mg/dL, hemoglobin concentrations up to 840 mg/dL, and triglyceride concentrations up to 1850 mg/dL. The spiked samples were analyzed, and results were compared against the original non-spiked serum sample.

#### Method comparison

De-identified serum samples from diabetic and non-diabetic patients were used to find the relationship between our LC–MS multiple reaction monitoring (MRM) method and the University of Missouri LC-MS SIM reference method (n = 47).

#### Quality assurance

The elution times of the target chromatographic peaks were monitored to ensure they fell within the 11.4–11.7 min window. The daughter ion b_11_ was used for quantitation and quality assurance, additional daughter ions b_14_, y_2_, and b_12_ were monitored. The ratios b_11_/y_2_, y_2_/b_14_, b_14_/b_12_, and b_12_/b_11_ were followed, with means (SD) of 2.62 (0.39), 1.09 (0.17), 0.87 (0.16), and 0.43 (0.10), respectively. Apart from completely failed experiments where peaks are entirely absent, deviations from established ratios are typically due to imperfections in the automatic integration by MultiQuant. This often includes areas adjacent to the target peak or incorrect assignment of the baseline, especially in samples with low C-peptide levels.

## Results and discussion

### Method development

**Fig. 1:**
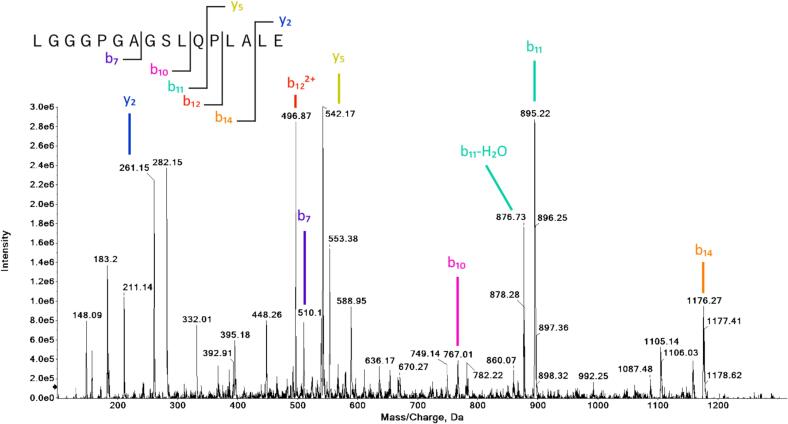
CID spectrum for LGGGPGAGSLQPLALE.

As C-peptide contains a certain number of Glu residues, Glu-C was the enzyme of choice. Information-dependent acquisition (IDA) for the digested C-peptide sample was performed to identify optimal precursors. Among the identified peptides − LGGGPGAGSLQPLALE, EAEDLQVGQVE, and DLQVGQVE − only LGGGPGAGSLQPLALE was chosen for quantitation, as it is the only labeled sequence from the commercially labeled C-peptide material. The collision-induced dissociation (CID) experiment performed on the peptide to identify the optimal MRM transitions revealed a spectrum rich in b ions (Fig. 1).

The digestion efficiency was evaluated by comparing the AUC of the target peptide released from labeled intact C-peptide with the AUC of the native target peptide spiked prior to LC-MS analysis. Although reducing the digestion duration from 24 to 2 h improved the yield, we proceeded with overnight digestion for the sake of maintaining the convenience of the overall workflow timeline. Digestion without DTT/IAA provided only 30 % lower of released peptide than with DTT/IAA, digestion without DTT/IAA but with heating denaturation performed similarly, and digestion with DTT/IAA/Zwittergent 3–16 was 17 % lower of released peptide. Since the denaturation and alkylation steps only moderately improve efficiency and the target C-peptide does not have disulfide bonds, we skipped these steps in our standard protocol to efficiently reduce the procedure time.

The vial material greatly affects recovery during evaporation, with C-peptide levels being much lower in polypropylene vials than in glass ones. We did not find that the addition of Zwittergent 3–16 improved recovery in any separation steps. It is crucial that all procedures involving pure C-peptide materials, including internal standards or CRM materials, be handled as solutions in 0.2 % HSA.

LC conditions were optimized to minimize carryover and reduce analysis time. The mass spectrometer parameters were optimized by direct infusion of pure synthetic peptide via a T-fitting connected to the LC–MS system. The optimal mass spectrometer parameters, such as voltage, collision energy (CE), temperature, Source Gas 1 (GS1), and GS2 were selected based on the intensities of MRM transitions.

The following MRM transitions were used for the native peptide: M^2+^ → b_11_^1+^ at 718.7/895.3 Da, M^2+^ → y_2_^1+^ at 718.7/261.3 Da, M^2+^ → b_12_^2+^ at 718.7/496.9 Da, and M^2+^ → b_14_^1+^ at 718.7/1176.6 Da. For the labeled peptide, the transitions were: M^2+^ → b_11_^1+^ at 721.7/895.3 Da, M^2+^ → y_2_^1+^ at 721.7/267.3 Da, M^2+^ → b_12_^2+^ at 721.7/496.9 Da, and M^2+^ → b_14_^1+^ at 721.7/1176.6 Da.

### Assay validation

#### Imprecision

Imprecision caused by sample preparation and LC-MS analysis was evaluated through inter-day and intra-day CV in two C-peptide serum samples with low and high C-peptide concentrations (low: 0.53 nmol/L, high: 2.88 nmol/L). The inter-day CV for the low C-peptide sample was 7.61 %, while the CV for the high C-peptide sample was 9.62 %. The intra-day CV for the low C-peptide sample was 8.93 %, while the CV for the high C-peptide sample was 7.12 %. The total CV for the low C-peptide sample was 11.73 %, while the CV for the high C-peptide sample was 11.97 %.

#### Stability

The stability of acidic digests with overnight storage at 4 °C resulted in a 2.1 % change for low C-peptide samples and a 7.9 % change for high C-peptide samples. Serum samples exhibited a 6.1 % change after 24 h at room temperature for low C-peptide samples and a 6.6 % change for high C-peptide samples. After two days of serum storage at 4 °C, changes of 2.4 % for low C-peptide and 6.3 % for high C-peptide were observed.

#### Limit of quantitation

**Fig. 2:**
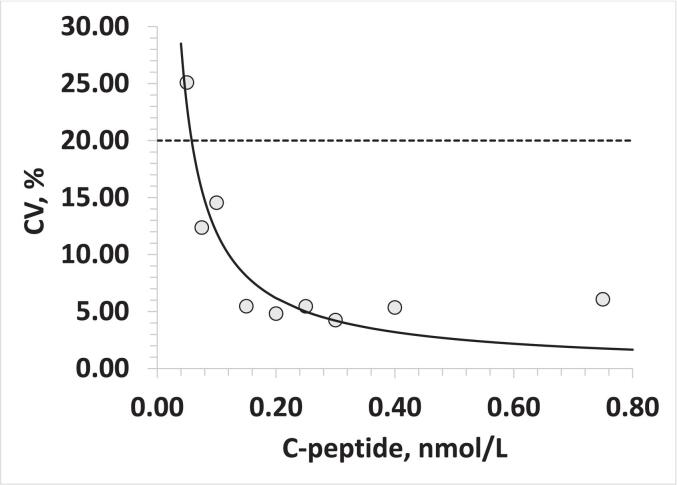
Lower Limit of Quantitation for C-Peptide. Imprecision of triplicate analyses at the low end of C-peptide concentration. The samples were prepared by spiking C-peptide CRM into horse serum.

**Fig. 3:**
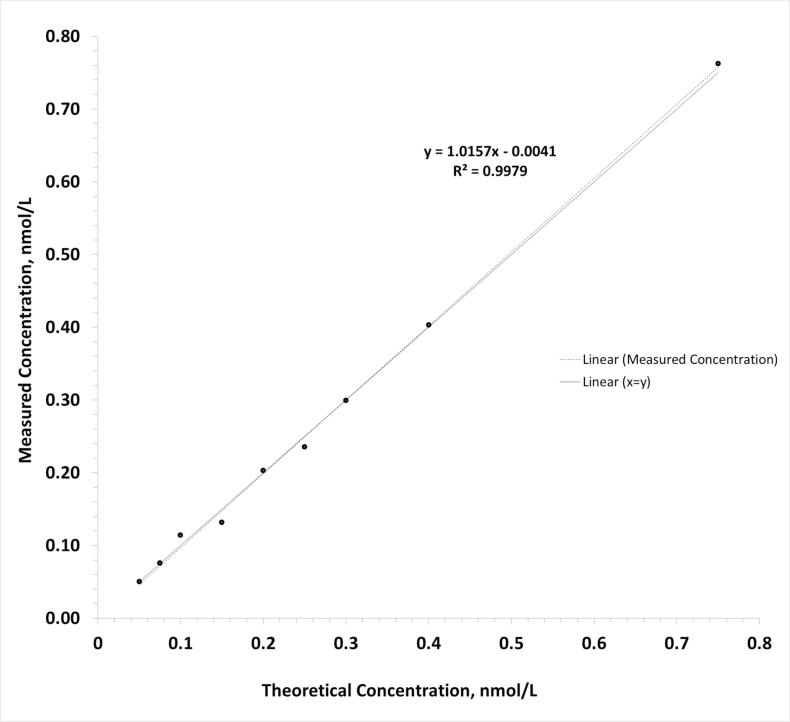
Accuracy of C-peptide measurements at low C-peptide concentrations. Samples were prepared by spiking C-peptide CRM material into horse serum, and C-peptide levels were quantified using the proposed approach.

The series of serum samples, prepared by spiking C-peptide CRM into the horse serum, were analyzed using the developed method. Using a CV of 20 % as the threshold, we found that the lower limit of quantification (LLOQ) for the digested C-peptide MRM method was 0.058 nmol/L (Fig. 2). The method demonstrated accuracy even at the low end of C-peptide concentration (Fig. 3).

#### Linearity

**Fig. 4:**
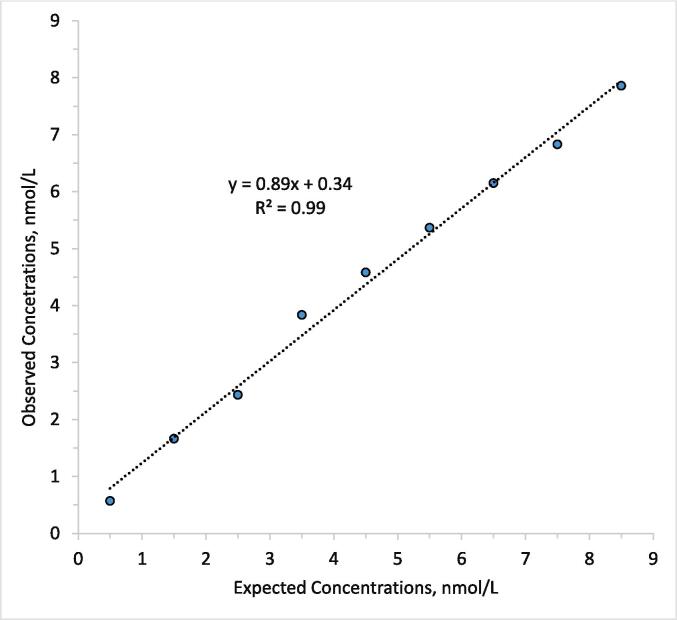
Linearity of Assay. A 10-point dilution series was prepared by spiking C-peptide CRM into C-peptide deficient serum of a diabetic individual and analyzed using the developed method. The dotted line represents the regression line.

The linearity of the assay was determined using a 10-point dilution series prepared by spiking NMIJ C-peptide CRM into C-peptide deficient serum from a type 1 diabetic individual. These dilutions were analyzed using the developed method. The assay was linear between 0.5 and 9.5 nmol/L (Fig. 4).

#### Interference

**Fig. 5:**
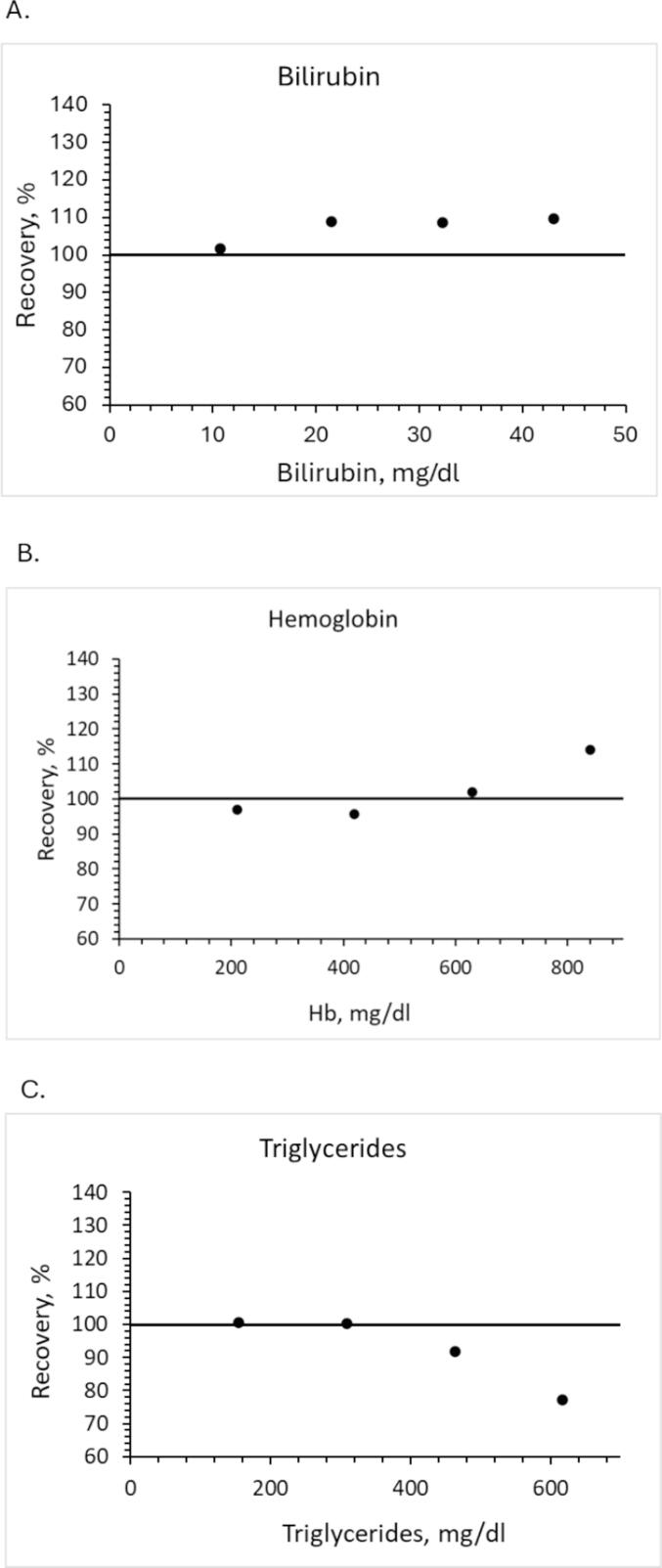
Interference study. C-peptide recovery at four concentrations of (A) bilirubin, (B) hemoglobin, and (C) triglycerides. Dilutions were prepared by spiking bilirubin, triglycerides, or hemoglobin into serum samples with a known C-peptide concentration.

The three most common clinical interferences tested were hemoglobin, triglycerides, and bilirubin. Hemoglobin and bilirubin were found not to cause any interference, as their recoveries were within 20 % of the threshold (Fig. 5). However, elevated levels of bilirubin led to 10x signal suppression and increased noise levels in the MRM chromatograms. Triglycerides, on the other hand, were found to cause interference at the highest level, resulting in a recovery of 77 % for concentrations of 617 mg/dL of triglycerides, respectively (Fig. 5).

#### Calibration

**Fig. 6:**
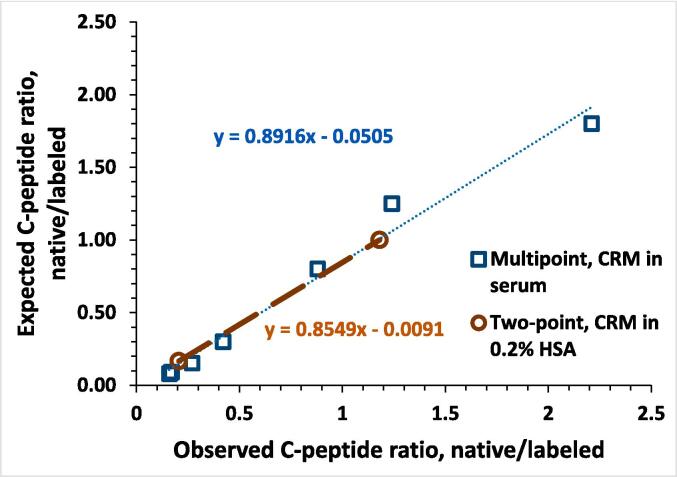
Comparison of linear calibration curves for different matrices. The two-point curve prepared by blending known amounts of C-peptide CRM with labeled C-peptide in 0.2% HSA, and the seven-point curve prepared by spiking labeled C-peptide and C-peptide CRM into serum from a diabetic individual with no detectable C-peptide.

We examined the impact of the matrix on the calibration curve. A two-point curve was created by mixing C-peptide CRM and C-peptide labeled material in a 0.2 % HSA solution. A seven-point calibration curve was created by spiking labeled C-peptide and C-peptide CRM into serum obtained from a type 1 diabetic individual with undetectable C-peptide levels. The matrix-matched seven-point calibration yielded results comparable to the two-point calibration curve, which was subsequently employed in the method for the sake of simplicity (Fig. 6).

#### Method comparison

**Fig. 7:**
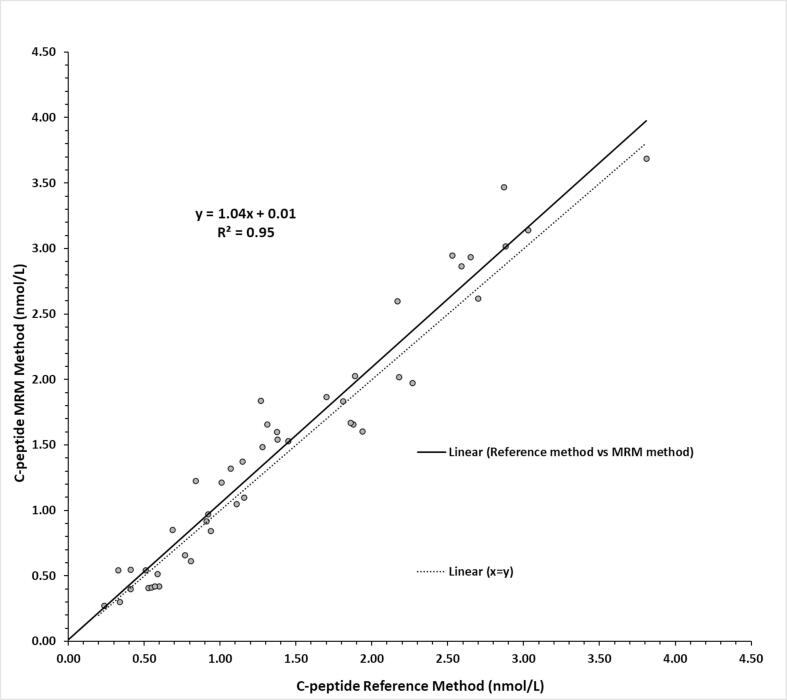
Comparison of C-peptide reference and MRM method. The comparison was done using 43 individual patient serum samples and four pooled serum samples routinely employed as quality controls.

Patient samples spanning a wide range of C-peptide were analyzed according to the general protocol. The C-peptide MRM method's correlation with the reference method is notably strong, as indicated by an R^2^ value approaching 1 and the proximity of the regression line to the identity line (Fig. 7). The difference between the methods, apart from their distinct enrichment protocols, lies in the fact that the reference method includes the quantification of the intact C-peptide molecule, whereas the newly developed method focuses solely on the quantification of the peptide released from Glu-C digestion of the C-peptide.

The C-peptide reference method separation was based on a sequence of elution on a strong anion exchange SP column followed by a strong cation exchange Q column. Mass spectrometry acquisition was performed in SIM mode for the + 3 charge state of the intact polypeptide chain in Q1. The method met all analytical standards necessary for a reference method when it was registered with JCTLM. Through the long-term implementation of a C-peptide standardization program in our laboratory, we have recognized the necessity for developing an auxiliary method that demonstrates robust analytical performance. The main reason is to monitor potential long-term method fluctuations due to factors like LC-MS platform changes, C-peptide internal standard variations, and C-peptide CRM adjustments. While we currently compare reference method with C-peptide immunoassays, their reliability is compromised by occasional vendor modifications, which are often undisclosed for proprietary reasons. These immunoassay changes can be unpredictable, underlining the importance of an alternative method to safeguard against these inconsistencies. An ideal auxiliary method should be based on mass spectrometry, but employ a distinct separation technique, measurement strategies, and measurands. We opted for MRM quantitation due to its high specificity, using Gluc-C digestion, as C-peptide contains glutamic amino acid residues, an approach previously proven to be efficient [17], [18]. Considering the distinct principles and measurements, this method can be considered independent. Comparison studies are particularly valuable for the C-peptide reference system, as illustrated by several scenarios. For example, the deamidation of C-peptide from glutamine (Gln) to glutamic acid (Glu) during long-term storage of primary or secondary reference materials may go undetected in SIM at unit resolution [19]. However, this change could become evident when using a Glu-C based digestion method. Additionally, when using patient samples, there remains a possibility of interference in the mass-to-charge window under SIM. Implementing an auxiliary orthogonal MRM approach can mitigate this risk.

Another purpose is to ensure that the current reference method provides accurate values for secondary reference materials. If the values obtained by both methods are within a 20 % variance, the average will be calculated. If the variance is higher, it will be scrutinized and discarded from the secondary reference material set if there is no improvement. Our current plan is to use both methods in the foreseeable future and to phase out the SIM method if we can demonstrate the long-term stability and reliability of the newly developed MRM method, as required for the standardization program. In addition, this method holds potential interest for other laboratories, as it can be utilized for the accurate quantitation of C-peptide. Although MRM is considered the gold standard for mass spectrometry-based quantitative analysis, the additional digestion step extends the turnaround time, but is expected to enhance specificity. While we have thoroughly described our method, replicating the exact implementation in other laboratories may not always be feasible due to the sophisticated nature of LC-MS techniques, which depend on numerous factors. Discrepancies can arise if different vendor supplies are used for Glu-C or LC columns. A more critical factor is the C-peptide internal standard, which may vary in isotopic enrichment or labeling patterns. Retention times can also depend on the column vendor, and even for the same vendor, they are prone to shift due to changes in the hydrophobicity of the column over time. Another important issue is the mass spectrometer platform utilized, as there are vendor-specific parameters that optimize performance. Thus, optimization of mass spectrometer parameters on different platforms is advisable. However, by following the provided calibration scheme with traceability to CRM, all variations should be compensated, allowing for comparable results. Unfortunately, certain characteristics, particularly retention times and daughter ion ratios, are prone to fluctuation depending on the technical implementation. While the new method has certain disadvantages compared to our reference method, such as being more time-consuming, involving additional procedural steps, and requiring the costly Glu-C enzyme, it remains viable for implementation in other laboratories for C-peptide quantitation. However, integrating the CRM calibration step is essential to ensure the true accuracy of the results and maintain a complete traceability chain [14].

## Conclusion

We have developed a mass spectrometry method for the quantitation of C-peptide in human serum. This method is based on Glu-C digestion and MRM quantitation and has demonstrated an analytical performance that can serve as an auxiliary method for the C-peptide reference method, ensuring the stability of the reference method.

## Ethics statement

Blood samples were collected (2015-2016) in accordance with IRB approval (University of Missouri, # 2003930) to serve as secondary calibrators for the C-peptide LC-MS reference method. The samples were de-identified, and no identifying information was used in the study. Informed consent was obtained.

## Funding sources

This work was supported by NIH/NIDDK (Grant Number 1UC4DK096587-01).

## CRediT authorship contribution statement

**Will Grothoff:** Writing – review & editing, Investigation, Data curation. **Ivan Khodakivskyi:** Writing – review & editing, Investigation, Data curation. **Aleks Shin:** Writing – review & editing, Investigation, Data curation. **Randie Little:** Writing – review & editing, Funding acquisition. **Shawn Connolly:** Writing – review & editing. **Kuanysh Kabytaev:** Writing – original draft, Methodology, Conceptualization.

## Declaration of competing interest

The authors declare that they have no known competing financial interests or personal relationships that could have appeared to influence the work reported in this paper.
